# Shared Medical and Environmental Risk Factors in Dry Eye Syndrome, Sjogren's Syndrome, and B-Cell Non-Hodgkin Lymphoma: A Case-Control Study

**DOI:** 10.1155/2019/9060842

**Published:** 2019-01-21

**Authors:** Hadas Ben-Eli, Doron J. Aframian, Eldad Ben-Chetrit, Dror Mevorach, Geffen Kleinstern, Ora Paltiel, Abraham Solomon

**Affiliations:** ^1^Braun School of Public Health and Community Medicine, Hadassah-Hebrew University of Jerusalem, Israel; ^2^Department of Ophthalmology, Hadassah-Hebrew University Medical Center, Jerusalem, Israel; ^3^Department of Optometry and Vision Science, Hadassah Academic College, Jerusalem, Israel; ^4^Department of Oral Medicine, Sedation and Maxillofacial Imaging and Sjogren's Syndrome Center, Hadassah-Hebrew University Medical Center, Jerusalem, Israel; ^5^Unit of Rheumatology, Hadassah-Hebrew University Medical Center, Jerusalem, Israel; ^6^Department of Internal Medicine, Hadassah-Hebrew University Medical Center, Jerusalem, Israel; ^7^Department of Health Sciences Research, Mayo Clinic, Rochester, MN, USA; ^8^Department of Hematology, Hadassah-Hebrew University Medical Center, Jerusalem, Israel

## Abstract

**Objectives:**

To assess whether there are shared exposures associated with Sjogren's syndrome (SS), dry eye syndrome (DES), and B-cell non-Hodgkin lymphoma (B-NHL), in order to determine whether they are etiologically related.

**Methods:**

In a clinic-based case-control study, 702 participants (91 SS, 120 DES, 211 (age and sex frequency-matched) controls, and 280 B-NHL cases) were recruited and interviewed regarding exposures, medical history, and family history.

**Results:**

Female predominance was noted in SS (ratio 9.2 : 1). Eye dryness was severest in SS compared to DES and controls (*P* < 0.001). Compared to controls, alcohol consumption was inversely associated with NHL, DES, and SS (odds ratio (OR) = 0.47, 95% confidence interval (CI): 0.31-0.71; OR = 0.54, 95% CI: 0.33-0.88; and OR = 0.26, 95% CI: 0.14-0.49, respectively), while a previous history of infection requiring hospitalization was positively associated with all three conditions: NHL (OR = 1.92; 95% CI: 1.23-2.99), DES (OR = 3.29; 95% CI: 1.97-5.47), and SS (OR = 4.74; 95% CI: 2.66-8.44). NHL patients were more likely to report first-degree relatives with hematologic cancer, while having first-degree relatives with an autoimmune disease (AID) was associated with SS (OR = 5.25; 95% CI: 2.59-10.63) and DES (OR = 3.55; 95% CI: 1.83-6.91) compared to controls.

**Conclusions:**

Some exposures are associated with all three conditions (such as an inverse association with alcohol consumption and a positive association with serious past infection), while a family history of AID appears to be shared by DES and SS, but not NHL subjects. Shared risk factors for all three conditions indicate possible mutual etiological pathways.

## 1. Introduction

In order to further our understanding of neoplastic transformation, it is important to understand the medical and environmental antecedents of disease. In this study, we explored whether environmental and medical exposures were shared among subjects with an autoimmune disease, a related condition, and B-cell lymphomas.

Dry eye syndrome (DES) is a common disorder, associated with older age [1] and female gender [2–4] as well as with environmental stimuli such as contact lens wear [5], use of air conditioners, occupations such as farming, outdoor labour [5], and work with visual display terminals [6]. Other risk factors associated with DES are African-American ethnicity [1], medical conditions such as diabetes (mainly type 2), autoimmune conditions, noninflammatory arthritic conditions, thyroid diseases, sleep apnoea [1, 3], and posttraumatic stress disorder (PTSD) [1]. Medications such as antihistamines, antianxiety, and antidepressants have been implicated [1].

Dry eyes and dry mouth are the cardinal features of Sjogren's syndrome (SS), an autoimmune disease (AID) with a strong female predilection [7–9]. A family history of SS in first-degree relatives is known to be associated with SS with odds ratios (ORs) ranging from 7 to 19 [10, 11], as well as with other AIDs such as rheumatoid arthritis [11]. Environmental risk factors found to be inversely associated with SS include smoking and previous blood transfusions [10]. Alcohol consumption was found to have a protective effect on AIDs such as systemic lupus erythematosus (SLE) [12], but this effect has not been reported in relation to SS.

Patients with SS have a markedly increased risk of developing B-cell non-Hodgkin lymphoma (B-NHL) [13–16], with ORs of 7.0 in registries [17, 18] to a relative risk (RR) of 18.9 in meta-analyses [13, 19] compared to the general population. Increased risks were consistently found for MALT lymphoma, marginal zone lymphoma (MZL), diffuse large B-cell lymphoma (DLBCL), and lymphoplasmacytic lymphoma (LPL) subtypes [20–22]. Risk factors for NHL include a history of AIDs [23], HCV seropositivity, and occupations such as painting and farm work [21]. Independent systemic and serological risk factors which may serve as predictors for NHL development in SS patients include salivary gland enlargement, lymphadenopathy, Raynaud's phenomenon, anti-Ro/SSA or/and anti-La/SSB positivity, RF positivity, monoclonal gammopathy, and C4 hypocomplementaemia [24]. Factors inversely associated with NHL from a large international consortium of case-control studies include alcohol consumption, recreational sun exposure, blood transfusions, and high socioeconomic status [21].

We performed a case-control study comparing medical, personal, and occupational history in these three diseases. We aimed to determine whether DES, SS, and B-NHL share common exposures in order to explore the possibility that they represent an etiologic continuum.

## 2. Methods

### 2.1. Design

This was a clinic-based case-control study.

### 2.2. Study Population

We included subjects > 18 years of age. SS patients were obliged to fulfil four out of six criteria of the American-European Consensus Group (AECG) classification [23]: symptoms of dry eyes, signs of dry eyes, symptoms of dry mouth, signs of dry mouth, a positive minor salivary gland biopsy with a focus core of ≥1/4 mm^2^, or the presence of anti-Ro/SSA or anti-La/SSB. Eligible NHL cases included newly diagnosed (<18 months since diagnostic biopsy) patients diagnosed in 2009-2014, with hematopathologist-confirmed diagnoses of marginal zone lymphoma (MZL) or diffuse large B-cell lymphoma (DLBCL and positive immune-staining for CD20 or other B-cell markers). The inclusion criteria for the DES group were as follows: subjects with complaints of dry eyes who had an Ocular Surface Disease Index (OSDI) questionnaire [25, 26] score of >25, positive Schirmer I test (without anaesthesia, <5 mm in 5 min), or break of fluorescein corneal dye in less than 5 sec in tear breakup time (TBUT) (which represents abnormal tear film stability and dry eye) [27], in at least one eye. Contact lens wearers were not excluded. Controls were healthy individuals free of SS and NHL and with no eye symptoms or OSDI < 25. Control subjects were sex and age (±3 years) frequency-matched to SS and DES subjects, in a way that a group of cases was matched to a group of controls with respect to these particular confounders. The severity of eye dryness was compared between the SS, DES, and control groups using the OSDI questionnaire.

We excluded subjects who had undergone eye surgery within five years preceding enrolment, those with chronic corneal infection, and subjects who did not understand the questionnaire languages (English, Hebrew, and Russian) or did not sign the informed consent form. We excluded controls who were first-degree relatives of NHL subjects. DES subjects had negative immune serology for autoantibodies anti-SSA/SSB.

### 2.3. Recruitment Methods

DES subjects were recruited from individuals consulting the eye clinic in our medical center with symptoms of dry eyes or from those responding to an advertisement in the medical center's clinics, a newspaper advertisement to the general public, or an e-mail to employees. DES diagnosis was performed by a cornea specialist, as detailed above. SS patients were diagnosed by a rheumatologist, a cornea specialist, or an oral medicine specialist and were recruited from the Rheumatology, Cornea, Oral Medicine, or Haematology Clinics in Hadassah Medical Center, a tertiary medical center. NHL cases and controls were recruited in a parallel study [28], which included 516 NHL patients with variable subtypes and 414 healthy controls. Controls were recruited among individuals accompanying NHL subjects to the clinic. These included spouses (comprising 11% of controls) or other consenting volunteers but, as noted, did not include first-degree relatives of NHL patients.

### 2.4. Exposure Assessment

All participants were interviewed using a previously validated questionnaire utilized in epidemiological studies of NHL [29–32] which includes items regarding environmental exposures, demographic parameters, medical history, and family history of cancer and autoimmune diseases (AIDs). NHL cases were instructed to refer all responses to exposures prior to disease onset. In addition, DES, SS, and control subjects scored their eye dryness severity by filling out the self-administrated OSDI questionnaire, which had undergone forward and back translation from English to Hebrew.

Ethnicity was classified into four main categories for participants who were of Jewish origin: Eastern European, West Asian, North African, or mixed. Each participant was scored according to the number of parents, grandmothers, and grandfathers born in the same world region. An ethnic group was assigned if the score was ≥3 for a particular region or “mixed” if the score was <3. Education was classified as “high” if the participant reported >12 years of schooling. The cutoff for grand multiparity or large sibship size was ≥5. Smoking, hair dye use, blood transfusions, practicing art as a hobby (such as painting or sculpting), and exposure to pets or large animals that grew in the participant's house were classified as ever vs. never. Participants were asked if they, themselves, were breast-fed, and only those who were sure of this exposure were classified as “yes.” Alcohol consumption was scored as positive if it occurred at least once a week, and participants were deemed to be physically active if physical activity took place >3 times a week consistently over the past 10 years. Occupation was attributed to the longest held occupation or by a job held for >5 years and was classified into main categories including a health worker, teacher, office worker, housewife/pensioner, painting, agriculture, or other. The housewife/pensioner category included participants who had held no other main occupation for at least five years. We questioned subjects regarding episodes of infection requiring hospitalization which occurred prior to the diagnosis of the studied disease, first-degree relatives with an AID or hematologic cancer, and a medical history of diseases (any hepatitis, infectious mononucleosis, allergy, asthma, eczema, and inflammatory bowel disease). Medical records of NHL, SS, and DES subjects were used to confirm the diagnosis of self-reported AID.

Our study was approved by the Institutional Helsinki Ethics Committee (study # HMO-0409-13). Participants gave signed informed consent, and questionnaire data were coded anonymously.

### 2.5. Statistical Analysis

Normally distributed continuous variables were presented by means with standard deviation, and differences between and among groups were tested with a *t*-test and one-way ANOVA. Frequencies of demographic characteristics, environmental exposures, and medical history of study participants were compared among the study groups using a *χ*^2^ test. Associations between cases of DES, SS, and B-NHL and shared exposures and occupations compared to controls were assessed by multinomial logistic regression, while controlling for potential confounders and covariates. We report associations using OR with 95% confidence intervals (CI). Variables entered into the multivariable models were chosen according to their importance in the current literature and statistical significance in preliminary univariate analysis and were based on a priori associations with the tested diseases. Participants with missing values did not enter the regression models. A 2-sided *P* value of 0.05 was considered statistically significant in all tests. Statistical analysis was done using SPSS 23.0 software (Chicago, IL, 60606-63070).

### 2.6. Power Calculation

A study including 91 SS and 211 controls provided a minimal power of 80%, to detect an odds ratio (OR) of 2.7 [1, 10] with *α* = 0.05, given the prevalence of an exposure factor among controls of 10%. (WinPepi 11.63 software).

## 3. Results

A total of 702 participants were recruited, including 91 subjects with SS, 120 with DES, and 211 age- and sex-matched healthy controls. The B-cell non-Hodgkin lymphoma (B-NHL) group comprised 280 cases, including 71 with MZL and 209 DLBCL subtypes. Response rates were ≥95% for all cases and 75% for controls.

The demographic characteristics of the study population are shown in Table 1. In the SS group, a strong female predominance was noted (9.2 : 1), and in the DES group, it was 6.7 : 1. The mean age of all participants was similar among the study groups, ranging from 52 to 58.7 years, except for the DES where participants were slightly younger. In all study groups, East European ancestry was the most common, but in the SS group, there were slightly higher proportions of subjects with West Asian and North African origin compared to the other three study groups. Most of the study subjects were married, but a high proportion of DES participants was single; most subjects had >12 years of education, with a slightly lower proportion of SS patients attaining this educational level. Missing values were noted in <1% of the interview data.

**Figure 1:**
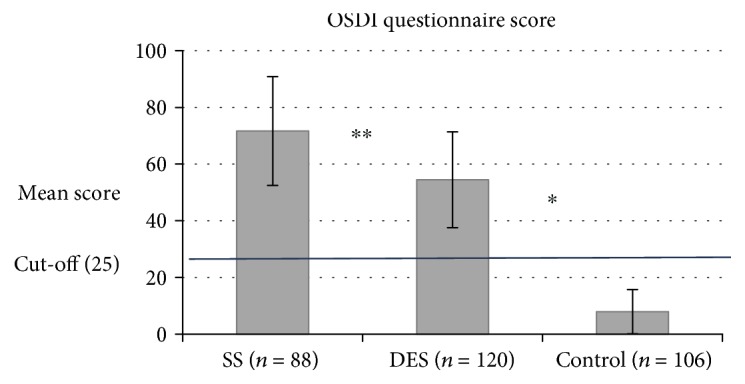
Severity of eye dryness by the OSDI questionnaire score. Cutoff score = 25. ^∗^Comparison between the 3 groups with a one-way ANOVA test: *P* < 0.001. ^∗∗^ SS vs. DES with an independent sample *t*-test: *P* < 0.001. Error bars: standard deviations.

Regarding the eye dryness severity scored by the OSDI questionnaire, we found that SS patients subjectively reported extreme eye dryness compared to DES subjects and controls with average scores of 71.6 ± 19.2, 54.4 ± 16.9, and 7.9 ± 7.8, respectively (*P* < 0.001) (Figure 1).

Table 2 summarizes the environmental exposures and medical history of the recruited participants and unadjusted analysis among groups. SS was found to be statistically significantly associated with a higher number of births and siblings (≥5), use of hair dye, art as a hobby, self-reported infection requiring hospitalization, asthma, inflammatory bowel disease, and having a first-degree relative with an AID compared to DES, NHL, and controls. NHL was associated with smoking (but note that most of the smokers were men, who represented a small proportion of the other study groups) and having been breast-fed, whereas DES participants more commonly reported hepatitis compared to other groups. While having a first-degree relative with an AID was statistically significantly more frequent in the SS and DES groups compared to NHL and controls (*P* < 0.001), having a first-degree relative with a hematopoietic cancer was more common in NHL subjects compared to DES, SS, and controls (*P* = 0.05).

Some associations, such as physical activity, art hobbies, having been breast-fed, hepatitis, inflammatory bowel disease, and asthma, lost their statistical significance in the multivariate analysis. We thus excluded these factors from further regression models. After adjusting for age, sex, and education (Table 3), we observed an inverse association of NHL, DES, and SS with alcohol consumption (OR = 0.47, 95% CI: 0.31-0.71; OR = 0.54, 95% CI: 0.33-0.88; and OR = 0.26, 95% CI: 0.14-0.49, respectively). Mixed ancestry was significantly associated with SS (OR = 3.19; 95% CI: 1.19-8.57) and DES (OR = 2.21; 95% CI: 1.00-5.1), and North African ethnicity with SS (OR = 2.27; 95% CI: 1.05-4.93) compared to controls. Subjects with NHL, DES, and SS were more likely to report infection requiring hospitalization preceding the disease diagnosis (OR = 1.92, 95% CI: 1.23-2.99; OR = 3.29, 95% CI: 1.97-5.47; and OR = 4.74, 95% CI: 2.66-8.44, respectively) than control subjects. Furthermore, having a first-degree relative with hematopoietic cancer was found to be associated with NHL (OR = 1.93; 95% CI: 1.02-3.67), while first-degree relative with an AID is more frequently seen in SS (OR = 5.25; 95% CI: 2.59-10.63) and DES (OR = 3.55; 95% CI: 1.83-6.91) compared to controls (Table 3).

We restricted our analysis of occupation to female SS patients and their matched controls only, since 90% of the SS group were women. Moreover, some of the DES participants were volunteers and hospital employees (*n* = 57) recruited using advertisements, and thus, analyses regarding occupation in DES recruits were of limited value. In a logistic regression model after adjustment for age and sex, SS was associated with a nonsignificantly increased odds ratio in health- and office-related occupations (OR = 1.44, 95% CI: 0.37-5.56; OR = 1.28, 95% CI: 0.41-4.00, respectively), while teaching or other occupations showed a nonsignificant negative association with SS compared to controls (OR = 0.76, 95% CI: 0.21-2.73; OR = 0.40, 95% CI: 0.08-1.90, respectively).

## 4. Discussion

The hypothesis of this study was that there may exist a continuum of risk that starts with a common disorder, DES, progressing through an AID, such as SS, ending eventually in B-cell neoplasms in susceptible individuals. We explored whether there are shared environmental exposures, medical history, and family history among subjects with these diseases compared to healthy controls in a case-control study.

As expected, in SS, we found a strong female to male ratio (9.2 : 1), which is consistent with the range previously reported, from 9 : 1 up to 20 : 1 in the fourth to fifth decades of life [7, 9]. DES cases also demonstrated a female predilection [1, 2]. In addition, the subjective symptom score for eye dryness among SS patients was extremely high, similar to mean scores reported in previous studies on SS [33, 34] and in line with the DEWS stage 4 severity definition (severe dry eye) [5, 35]. The OSDI mean score of DES subjects was slightly lower than that of SS patients and was classified as grade 3 by DEWS. As expected, the controls' mean scores were much lower, beneath the cutoff point of moderate dry eye (<25) and even lower than the score of no dryness (<12) classified as “no disability” [35].

A history of hematologic cancer among first-degree relatives was reported in other studies as a risk factor for NHL, with very similar ORs to the one we found (OR = 1.48 − 1.79) [20, 21, 28]. Interestingly, while having a first-degree relative with hematologic cancer was found to be a risk factor for NHL but not for SS or DES, having a first-degree relative with an AID was a risk factor for SS and DES but not for NHL. Thus, there appears to be some specificity of family history to disease status. Priori and colleagues reported on a sevenfold increased OR for SS in subjects with a first-degree relative with an AID (OR = 7.4; 95% CI: 2.8-20.1) [10], as compared to the fivefold increase we found in SS subjects and threefold in DES subjects compared to controls. In another historical cohort study, a relative risk (RR) of 18.99 (95% CI: 9.76-36.93) was reported for SS in siblings of patients with SS, 11.31 (95% CI: 8.34-15.33) in offspring, and 12.46 (95% CI: 9.34-16.62) in parents [11].

The mechanism by which lymphoma develops on a background of B-cell activating immune disease is not fully known, but it has been suggested that several infections including viruses, together with genetic aberration, cytokine, and chemokine release, may initiate autoimmune stimulation, lymphocyte homing, and chronic inflammation [18, 24, 36, 37], involving the innate and acquired immune systems. We found that self-reported serious infection requiring hospitalization was strongly associated with DES, SS, and NHL. These associations suggest these three diseases may have an underlying innate or acquired immune system disturbance resulting in decreased resistance to pathogens. They also raise the possibility of serious infection triggering disease initiation.

An interesting finding in our study was the association between SS and North African ancestry and between SS and DES and mixed ethnicities. Several studies on Turkish [38], Egyptian [39], Tunisian [40], and Moroccan [41] subjects have reported that these ethnic groups have a high proportion of sicca signs and symptoms, SS manifestations, and genetic susceptibility to SS compared to other populations. Ethnic differences have also been explored by Tran et al. [42], who reported the differences in the severity of eye dryness between 180 Asian and 215 non-Asian patients. They found higher corneal staining and eye dryness symptom scores among Asian subjects compared to non-Asians (*P* < 0.001, 42). In our study, Asian ancestry refers mainly to patients from West Asia: Iran, Iraq, Yemen, etc. The recent review of Brito-Zerón et al.summarized the influence of ethnicity on SS and its phenotypic expression [22]. The authors reported a twofold higher risk for SS in non-European ancestry compared to European and Asian subjects, similar to our current study findings, while further data from a large cohort reported on the early age diagnosis of SS and increased risk for systemic disease in African-American compared to White patients and decreased risk for sicca syndrome in Asian patients [22].

In the current study, we included cases with MZL and DLBCL, the two NHL subtypes that are most commonly associated with SS, together with lymphoplasmacytic lymphoma (LPL) [16, 21, 43]. As in several studies on NHL, we found that alcohol consumption is negatively associated with NHL [20, 21, 28] (OR = 0.47; 95% CI: 0.34-0.63). A novel finding in our study was that this inverse association with alcohol was also found to be statistically significant not only in NHL as was previously reported but also in DES and SS cases compared to controls. In a large meta-analysis of prospective studies that included 195,029 participants and 1,878 cases of the AID rheumatoid arthritis (RA), it was reported that low to moderate alcohol consumption has a preventive effect on RA development (relative risk = 0.86; 95% CI: 0.78-0.94), with a nonlinear dose response [41]. Moderate alcohol consumption has been described as having benefits to the immune system. The mechanism of an immune-enhancing effect of moderate alcohol consumption is postulated to be via decreased production of inflammatory mediator levels, such as TNF*α* and IL-1*β*, reduced plasma levels of inflammation markers fibrinogen, IL-1*α*, and soluble C-reactive protein, and decreased expression of endothelial adhesion molecules such as VLA4 and LFA-1 [42]. Immune-boosting effects of moderate alcohol consumption are also associated with increases in plasma antioxidant levels and with modulation in a dose-dependent expression of Toll-like receptors (TLRs) that promotes inflammatory responses with activation of NF*κ*B in an intracellular signal transduction cascades [42]. While immune modulation by alcohol may be a plausible mechanism for the observed association, on the other hand, this association may be due to differences in other characteristics of alcohol drinkers and nondrinkers, rather than a causal effect [20].

It was also shown that SS patients had lower education levels, followed by the NHL subjects, with the highest levels on the DES group (57.1%, 64.7%, and 75.8%, respectively, *P* = 0.01). In contrast to a report on negative associations between NHL and socioeconomic status (SES), measured by years of education (OR = 0.88; 95% CI: 0.83-0.93) [18], on multivariate analysis, we found a nonsignificant association between SES and NHL, SS, or DES. Yet the lack of postsecondary education may be a proxy for a lower socioeconomic status [21], which was found to be in this study a risk factor for SS. Biased recruitment of DES patients may have contributed to apparent education disparities.

We also found that a high proportion of DES subjects was single and younger compared to other participants. The explanation for this may lie in our recruitment method, which brought about 47% of the volunteer DES participants who responded to a published advertisement. On the other hand, this may also indicate that DES may be more common among young adults exposed to contact lens wear [5] or the frequent use of computers [6].

Similarly to our results, SS was found to be associated with a high number of births, a finding which was previously reported in a case-control study with an OR of 2.5 (95% CI: 1.3-4.7) [10]. Smoking and previous blood transfusion were also reported in the same study, as we found in the current one, to have a nonsignificant inverse association with SS [10], so they are probably not important factors in its pathogenesis.

We did not find any strong or statistically significant associations between occupation and SS. Others have reported an inverse association with teaching as a profession (OR = 0.86; 95% CI: 0.77-0.95) [21] as well as nonsignificant association between being a medical doctor (OR = 1.24; 95% CI: 0.74-2.10) [20] and disease status. A study among Swedish SS patients tried to investigate occupation as a risk factor for SS, but since the maternal occupation most often stated was “housewife,” insufficient data was obtained for meaningful analyses [43].

As in any questionnaire-based case-control study, recall bias may influence the results. As opposed to incident cases of NHL recruited in this study, SS patients were prevalent cases recruited from a tertiary clinic, which may result in selection bias. However, rheumatic diseases are characterized by vague and nonspecific symptoms and no definitive biomarker, and the diagnosis can be long after the beginning of the disease [7], making it impossible to pinpoint the time of the onset of disease. We also recruited DES patients attending a tertiary cornea clinic, but these were supplemented with patients from the community who responded to an advertisement. A significant proportion of them (47.5%) was hospital employees, who may differ from the general population in terms of exposure and self-reports of past illnesses. In order to reduce misclassification, we verified the symptoms of DES subjects with objective clinical tests and with a symptomatology score. The controls were individuals accompanying patients to a haematology clinic, some of whom may have shared environmental exposures to the NHL cases, although this would have biased the results toward the null hypothesis. Another limitation of this work may be residual confounding, though we adjusted for education, gender, and age in our models. The sample size was limited and affected the power of our analyses. SS is a rare disease, and pooling data through consortia would be beneficial to further understand its etiology. Finally, DES is a heterogeneous condition, in terms of both etiology and severity, and this study was not designed or powered to differentiate among DES subcategories.

The hypothesis that SS may begin as DES and then develop into B-NHL was not fully supported by the study findings, but we found shared exposures and risk factors to these diseases compared to healthy controls, suggesting that chronic inflammation and autoimmunity may be at the basis of this continuum. Further focus on risk factors for DES, SS, and NHL and related exposures may contribute to the understanding of their pathogenesis.

## 5. Conclusions

SS is well known to be associated with an increased lifetime risk of B-NHL, and we wished to explore whether common factors may influence the development of DES, SS, and B-NHL. Our findings demonstrate that some lifestyle factors (e.g., lack of alcohol consumption), as well as severe infections requiring hospitalization, are common to all three conditions. Other factors were specific to one or two of these disorders, such as North African and mixed ethnicity for SS, reported here for the first time as a risk factor for SS. Also, having a first-degree relative with an AID was specifically associated with DES and SS, while reporting first-degree relatives with hematologic cancer related only to NHL. Clinical parameters, such as eye dryness severity among SS patients, differ from DES subjects and healthy controls. This multidisciplinary study exploring environmental and medical exposures in three possibly related diseases may shed light on their pathogenesis. A further work is required to understand events and exposures leading to B-cell NHL in autoimmune disease.

## Figures and Tables

**Table 1:** Demographic characteristics of study population.

Characteristic	Control (*N* = 211) *N* (%)	DES (*N* = 120) *N* (%)	SS (*N* = 91) *N* (%)	B-NHL (*N* = 280) *N* (%)	*P* (*χ*^2^)
Female gender	168 (79.6)	81 (67.5)	84 (92.3)	143 (51.1)	**<0.001**
Mean age (SD)	54.5 (14.2)	52.05 (15.1)	56.8 (12.8)	58.7 (16.2)	0.29
Ethnicity:					**0.04**
Eastern European	130 (61.6)	67 (55.8)	36 (39.6)	160 (57.1)	
West Asian	35 (16.6)	20 (16.7)	22 (24.2)	47 (16.8)	
North African	32 (15.2)	14 (11.7)	22 (24.2)	47 (16.8)	
Mixed	14 (6.7)	19 (15.8)	11 (12.1)	26 (19.2)	
Marital status:					**0.01**
Single	6 (2.8)	13 (10.8)	4 (4.4)	19 (6.8)	
First marriage	182 (68.3)	89 (74.2)	69 (75.8)	181 (64.6)	
Second marriage or more	10 (4.7)	4 (3.3)	4 (4.4)	17 (6.1)	
Divorced or separated	6 (2.8)	9 (7.5)	9 (9.9)	32 (11.4)	
Widowed	2 (0.9)	5 (4.2)	5 (5.5)	29 (10.4)	
Education > 12 yrs.	153 (72.5)	91 (75.8)	52 (57.1)	181 (64.7)	**0.01**
Number of births ≥ 5	33 (15.6)	12 (10.0)	22 (24.2)	58 (20.7)	**0.02**
Number of siblings ≥ 5	48 (22.7)	32 (26.7)	37 (40.7)	78 (27.9)	**0.01**

DES = dry eye syndrome; SS = Sjogren's syndrome; B-NHL = B-cell non-Hodgkin lymphoma; *χ*^2^ = chi-square test; SD = standard deviations.

**Table 2:** Environmental exposures and medical history of study population-unadjusted analysis.

Characteristic	Control (*N* = 211) *N* (%)	DES (*N* = 120) *N* (%)	SS (*N* = 91) *N* (%)	B-NHL (*N* = 280) *N* (%)	*P* (*χ*^2^)
Health behaviour:					
Ever smoked	91 (43.2)	52 (43.3)	30 (33)	142 (50.7)^a^	**0.01**
Alcohol consumption	123 (58.2)	58 (48.7)	25 (27.7)	127 (45.8)	**<0.001**
Hair dye (ever)	141 (66.8)	71 (59.2)	72 (79.1)	101 (36.1)^b^	**<0.001**
Physical activity (>3 times per week)	73 (34.6)	50 (41.7)	39 (42.9)	120 (42.9)	**0.008**
Art as a hobby	57 (27.0)	41 (34.2)	45 (49.5)	64 (22.9)	**<0.001**
Medical history:					
Blood transfusion (ever)	42 (19.9)	16 (13.3)	22 (24.7)	51 (18.2)	0.20
Self-reported hospitalization for infection	44 (20.8)	55 (45.8)	50 (54.9)	91 (32.5)	**<0.001**
Any hepatitis	27 (12.8)	20 (16.6)	11 (12.08)	36 (12.9)	**0.004**
Infectious mononucleosis	27 (12.8)	21 (17.5)	15 (16.5)	28 (10.0)	0.08
Allergy	58 (27.5)	46 (38.3)	38 (41.8)	84 (30.0)	0.26
Asthma	25 (11.8)	21 (17.5)	20 (22.0)	26 (9.3)	**0.03**
Eczema	27 (12.8)	25 (20.8)	18 (19.8)	42 (15.0)	0.14
Inflammatory bowel disease	3 (1.4)	10 (8.3)	20 (22.0)	11 (3.9)	**<0.001**
Personal history of AID	20 (9.5)	11 (9.2)	91 (100)	43 (15.3)	**<0.001**
Occupation (females only)					
Health worker	21 (12.5)	29 (35.8)	9 (10.7)	12 (8.4)	
Teacher	27 (16.1)	8 (9.9)	17 (20.2)	30 (20.9)	
Office worker	91 (54.2)	30 (37.1)	35 (41.7)	47 (32.8)	
Agriculture	1 (0.6)	0 (0)	2 (2.4)	4 (2.8)	
Housewife/pensioner	16 (9.6)	0 (0)	7 (8.3)	24 (16.8)	
Other	5 (2.9)	1 (1.3)	10 (11.9)	9 (6.3)	**<0.001**
Other exposures:					
1st-degree relative with AID	19 (9)	31 (25.8)	33 (36.2)	13 (4.7)	**<0.001**
1st-degree relative with hematopoietic cancer	13 (6.1)	11 (9.1)	5 (5.5)	38 (13.9)	**0.05**
Breast-fed	124 (58.8)	72 (60.0)	52 (57.1)	174 (62.1)	**0.009**
In-house pets/large animals	135 (64.0)	80 (66.7)	55 (60.4)	158 (56.4)	0.22

^a^Number of NHL women who have ever smoked: 54 (19.2%). ^b^Number of NHL women who have dyed their hair: 94 (33.5%). *χ*^2^ = chi-square test; DES = dry eye syndrome; SS = Sjogren's syndrome; B-NHL = B-cell non-Hodgkin lymphoma; AID = autoimmune disease.

**Table 3:** Risk factors—multinomial regression model.

Risk factor	Control	DES		SS		NHL	
	OR	OR	95% CI	OR	95% CI	OR	95% CI
Ethnicity:							
North African	1	0.69	0.32-1.46	**2.27**	1.05-4.93	0.88	0.49-1.58
West Asian	1	1.06	0.54-2.10	1.68	0.79-3.58	0.97	0.56-1.69
Mixed	1	**2.21**	**1.00-5.14**	**3.19**	**1.19-8.57**	1.45	0.68-3.10
Eastern European	1	Ref		Ref		Ref	
Smoking (yes vs. no)	1	0.90	0.55-1.47	0.76	0.42-1.35	1.17	0.78-1.75
Alcohol consumption (yes vs. no)	1	**0.54**	**0.33-0.88**	**0.26**	**0.14-0.49**	**0.47**	**0.31-0.71**
Hospitalization for infection (yes vs. no)	1	**3.29**	**1.97-5.47**	**4.74**	**2.66-8.44**	**1.92**	**1.23-2.99**
1st-degree relative with AID (yes vs. no)	1	**3.55**	**1.83-6.91**	**5.25**	**2.59-10.63**	0.59	0.27-1.27
1st-degree relative with hematopoietic cancer (yes vs. no)	1	1.31	0.57-3.00	0.65	0.22-1.96	**1.93**	**1.02-3.67**

Reference group: control. Adjusted for age (continuous), gender, and education. AID = autoimmune disease; CI = confidence interval; OR = odds ratio; DES = dry eye syndrome; SS = Sjogren's syndrome; B-NHL = B-cell non-Hodgkin lymphoma; Ref = reference category.

## Data Availability

The data used to support the findings of this study are included within the article.

## Additional Points

*Key Messages*. Several risk factors are common to Sjogren's syndrome, dry eye syndrome, and non-Hodgkin lymphoma. Familial autoimmunity is commonly reported in SS and DES, whereas familial lymphoma is specific to NHL patients.
